# The theory on and software simulating large-scale genomic data for genotype-by-environment interactions

**DOI:** 10.1186/s12864-021-08191-z

**Published:** 2021-12-05

**Authors:** Xiujin Li, Hailiang Song, Zhe Zhang, Yunmao Huang, Qin Zhang, Xiangdong Ding

**Affiliations:** 1 Guangdong Provincial Key Laboratory of Waterfowl Healthy Breeding, College of Animal Science & Technology, Zhongkai University of Agriculture and Engineering, Guangdong 510225 Guangzhou, People’s Republic of China; 2 Key Laboratory of Animal Genetics and Breeding of the Ministry of Agriculture and Rural Affairs, National Engineering Laboratory for Animal Breeding, College of Animal Science and Technology, China Agricultural University, 100193 Beijing, China; 3 Guangdong Provincial Key Lab of Agro-animal Genomics and Molecular Breeding, College of Animal Science, South China Agricultural University, Guangzhou, 510642 People’s Republic of China; 4 Shandong Provincial Key Laboratory of Animal Biotechnology and Disease Control and Prevention, College of Animal Science and Veterinary Medicine, Shandong Agricultural University, 271001 Taian, China

**Keywords:** Data simulation, Genotype-by-environment interaction, Threshold trait, GPOPSIM2.0

## Abstract

**Background:**

With the emphasis on analysing genotype-by-environment interactions within the framework of genomic selection and genome-wide association analysis, there is an increasing demand for reliable tools that can be used to simulate large-scale genomic data in order to assess related approaches.

**Results:**

We proposed a theory to simulate large-scale genomic data on genotype-by-environment interactions and added this new function to our developed tool GPOPSIM. Additionally, a simulated threshold trait with large-scale genomic data was also added. The validation of the simulated data indicated that GPOSPIM2.0 is an efficient tool for mimicking the phenotypic data of quantitative traits, threshold traits, and genetically correlated traits with large-scale genomic data while taking genotype-by-environment interactions into account.

**Conclusions:**

This tool is useful for assessing genotype-by-environment interactions and threshold traits methods.

## Background

Access to dense single nucleotide polymorphism (SNP) markers across the genome has created the opportunity for finely identifying quantitative trait loci (QTLs) through genome-wide association studies (GWASs) and accurately predicting genetic values through genomic selection (GS) for economically important traits in animal and plant breeding [1–3]. The related methodologies are developing rapidly, and generally, these new methods need to be evaluated through computer simulation before implementation with real data. Simulation is a cost-effective way to assess new approaches for GWASs and GS, and many simulation tools have been developed accordingly.

Genotype-by-environment (G-by-E) interactions have long been a topic of research interest. Generally, models applied to genetic evaluations do not consider G-by-E interactions, resulting in reductions in genetic gains. Many studies have reported that models accounting for G-by-E interactions improved the accuracy of estimates of genetic parameters and breeding values for complex traits [4–6]. Meanwhile, an increasing number of investigations on the detection of G-by-E interactions has been carried out in GWASs, although detecting such interactions is inherently more difficult than determining additive genetic effects [7, 8]. Compared to those needed for traditional GWASs, a larger sample size and more environmental levels for individual records are required to interpret G-by-E interactions, and it is obviously challenging to find such samples. Simulation is a key step in providing simulated data with large-scale genome SNP markers for assessing algorithms and methods for detecting G-by-E interactions. However, most of the developed software tools cannot provide this functionality [9, 10]. This greatly hinders the development of studies on G-by-E interactions in the framework of GWASs and GS.

Many traits of economic importance, such as litter size of large mammals, degree of calving difficulty and resistance to disease, show a discrete character of phenotypes, and are defined as threshold traits [11]. Due to phenotypic characters of threshold traits, the GWASs and GS methods for continuous traits are not appropriate for such kind of traits [12]. The threshold model, which links an underlying continuity with the outward phenotype, has been recommended for threshold trait analysis [11–14]. High-quality simulation data is a good option to carry out the investigation of GWASs and GS methods and breeding programs for threshold traits.

Previously, we developed the simulation tool GPOPSIM, which can simulate large-scale genomic data including population structure, polymorphic markers and multiple quantitative traits based on the mutation-drift equilibrium model [15]. The objective of this article is to propose a theory on the simulation of large-scale genomic data with G-by-E interactions and add this new function to our developed tool GPOPSIM. In addition, the simulation of threshold traits is also added.

## Implementation

### Theory

Generally, G-by-E interactions are analysed by a multi-trait model or a reaction norm model [11]. If environmental factors are categorized, phenotypes in different environments are treated as genetically separate traits, and genetic correlations between environments are a measure of the existence of G-by-E interactions [16, 17]. If environmental factors are quantified and are described by a continuous variable, we analyse G-by-E interactions using the reaction norm model in which phenotypes generally have a linear relationship with the continuous environmental variable, and breeding values and genetic parameters change gradually along this continuous variable [4, 18]. Because the reaction norm mode is widely used in G-by-E interactions, and GPOPSIM includes the function of multi-trait model, the reaction norm model was used to simulate a phenotypic value and an environmental value. Different from the AlphaSimR [19], the more complex reaction norm model accounting for heterogeneous residual variance is used here:

$$\mathrm{y}={\alpha}_0+{\alpha}_1\ast c+{e}_0+{e}_1\ast c,$$

where y is the phenotypic value; *c* is the environmental value; *α*_0_ and *α*_1_ are the zero- and first-order random regression coefficients of the breeding value on *c*, respectively; and *e*_0_ and *e*_1_ are the zero- and first-order random regression coefficients of the residual effect on *c*, respectively.

The environmental value *c* is further divided into two components:

$$\mathrm{c}=\upbeta +\upepsilon,$$

where β is the random genetic effect and ϵ is the random residual effect.

We assume that *α*_0_, β and *α*_1_ are affected by all QTLs simultaneously, and these three effects of each QTL are drawn from a multivariate normal distribution with a vector of means 0 and the variance-covariance structure $$\left[\begin{array}{ccc}{\sigma}_{\alpha_0}^2& {\sigma}_{\alpha_0\upbeta}& {\sigma}_{\alpha_0{\alpha}_1}\\ {}{\sigma}_{\alpha_0\upbeta}& {\sigma}_{\upbeta}^2& {\sigma}_{\upbeta {\alpha}_1}\\ {}{\sigma}_{\alpha_0{\alpha}_1}& {\sigma}_{\upbeta {\alpha}_1}& {\sigma}_{\alpha_1}^2\end{array}\right]$$. The genetic variance of each QTL is computed by 2 *p*_*i*_(1 − *p*_*i*_)*m*_*i*_, where *p*_*i*_ is the frequency of one allele of the i^th^ QTL and *m*_*i*_ is the effect of the i^th^ QTL for *α*_0_, β or *α*_1_. Then, the substitution effects are rescaled to ensure total variances ${\sigma}_{\alpha_0}^2$, ${\sigma}_{\upbeta}^2$ and ${\sigma}_{\alpha_1}^2$ for *α*_0_, β and *α*_1_, respectively. ${\sigma}_{\alpha_0\upbeta}$, ${\sigma}_{\alpha_0{\alpha}_1}$ and ${\sigma}_{\upbeta {\alpha}_1}$ are recalculated by using the scaled substitution effects of QTLs. The values of *e*_0_, *e*_1_ and ϵ for each individual are sampled from a multivariate normal distribution with a vector of means 0 and the variance-covariance structure $$\left[\begin{array}{ccc}{\sigma}_{e_0}^2& {\sigma}_{e_0{e}_1}& {\sigma}_{e_0\upepsilon}\\ {}{\sigma}_{e_0{e}_1}& {\sigma}_{e_1}^2& {\sigma}_{e_1\upepsilon}\\ {}{\sigma}_{e_0\upepsilon}& {\sigma}_{e_1\upepsilon}& {\sigma}_{\upepsilon}^2\end{array}\right]$$.When we set β, ${\sigma}_{e_0\upepsilon}$ and ${\sigma}_{e_1\upepsilon}$ to zero, the phenotypic value y and the environmental value *c* do not have a genetic relationship. Moreover, we can also generate the phenotype y and the environmental value *c* through the model y = *α*_0_ + *α*_1_ ∗ *c* + *e*_0_ without accounting for heterogeneous residual variance.

### Design

**Fig. 1:**
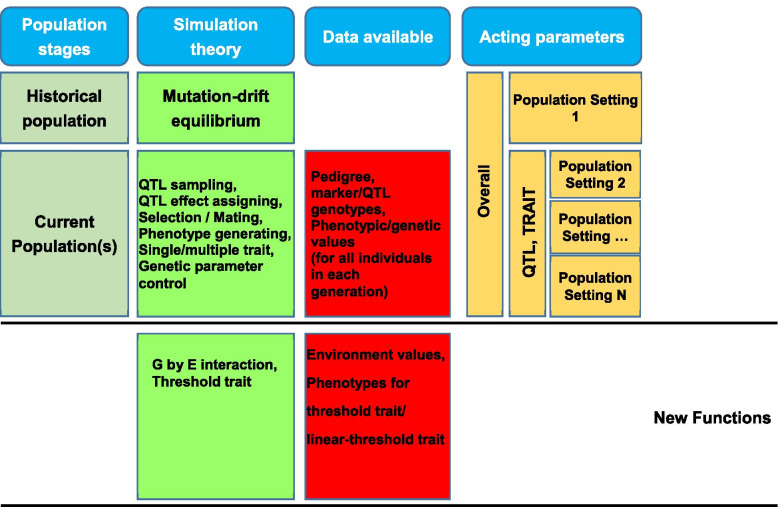
Workflow and parameter setting in GPOPSIM2.0

A parameter file is required to run GPOPSIM2.0 software. We specified various parameters for the simulation in this file. The parameter settings influence the historical population and the population structure, pedigree structure and genome structure of the current populations (Fig. 1). The simulation of populations starts with one historical population, and then one or more current populations are generated. The genome structure is clearly defined with related parameters, such as the number of chromosome, markers and QTLs. We create the polymorphic markers and the linkage disequilibrium among markers in the historical population. The true breeding value (TBV) of one individual is defined as the cumulative effect across all true QTLs, while the phenotypic value is generated by adding the TBV with a random residual error. Two or multiple genetically correlated quantitative traits can be also simulated. More details are described in our previous study [15].

For the simulation of a G-by-E interaction, ${\sigma}_{\alpha_1}^2$ is set in the parameter file to control the extent of the interaction, while other parameters (${\sigma}_{\alpha_0}^2$, ${\sigma}_{\upbeta}^2$, ${\sigma}_{\alpha_0\upbeta}$, ${\sigma}_{\alpha_0{\alpha}_1}$, ${\sigma}_{\upbeta {\alpha}_1},{\sigma}_{e_0}^2$, ${\sigma}_{e_1}^2$, ${\sigma}_{\upepsilon}^2$, ${\sigma}_{e_0{e}_1}$, ${\sigma}_{e_0\upepsilon}$ and ${\sigma}_{e_1\upepsilon}$) are fixed in the program. This can simplify the simulation parameters for the G-by-E interaction. The pseudo TBVs of an individual for *α*_0_, β or *α*_1_ are its QTL effects multiplied by genotypes, and then the means of the pseudo TBVs are scaled to 0. Finally, the environmental value c of each individual is obtained by adding the cumulative effect across all QTLs for β with the residual ϵ, and then the phenotype y of each individual is generated through the model y = *α*_0_ + *α*_1_ ∗ *c* + *e*_0_ + *e*_1_ ∗ *c* or the model y = *α*_0_ + *α*_1_ ∗ *c* + *e*_0_ without accounting for heterogeneous residual variance.

Additionally, threshold traits can be simulated by GPOPSIM2.0, which lies in the idea that discontinuous characters have an underlying continuity liability (i.e., a continuous phenotype), and threshold values divide the liability into discontinuity, resulting in some kinds of visible expression [11]. It is assumed that the liability follows a normal distribution, and the incidence values set in the parameter file are used to calculate the single-tailed normal deviations, i.e., threshold values.

### Source code and software availability

The GPOPSIM2.0 program is written in Fortran 90, and the source code is available free online. Executable files are currently performed on Windows and Linux platforms. GPOPSIM2.0 is free of charge for all users, and no licence is required (https://github.com/SCAU-AnimalGenetics/GPOPSIMv2). GPOPSIM2.0 can now simulate one or more independent/correlated quantitative traits, one or more independent/correlated threshold traits, genetically correlated quantitative-threshold traits and G-by-E interactions. The format of the input and output files is the same as that in GPOPSIM1.0.

## Results and discussion

**Fig. 2:**
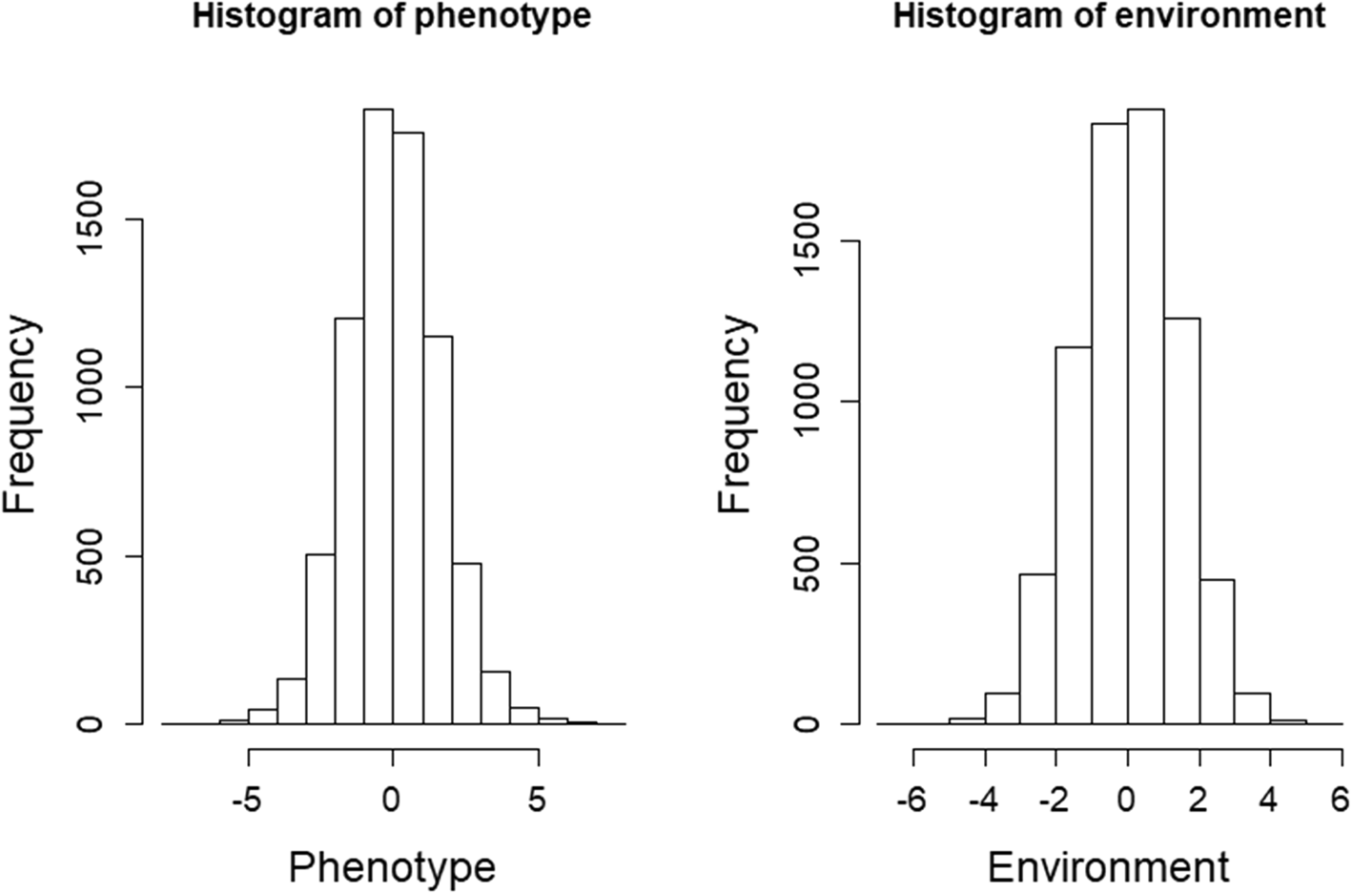
The distributions of phenotypic and environmental values for one replicate of simulated data including the G-by-E interaction

**Fig. 3:**
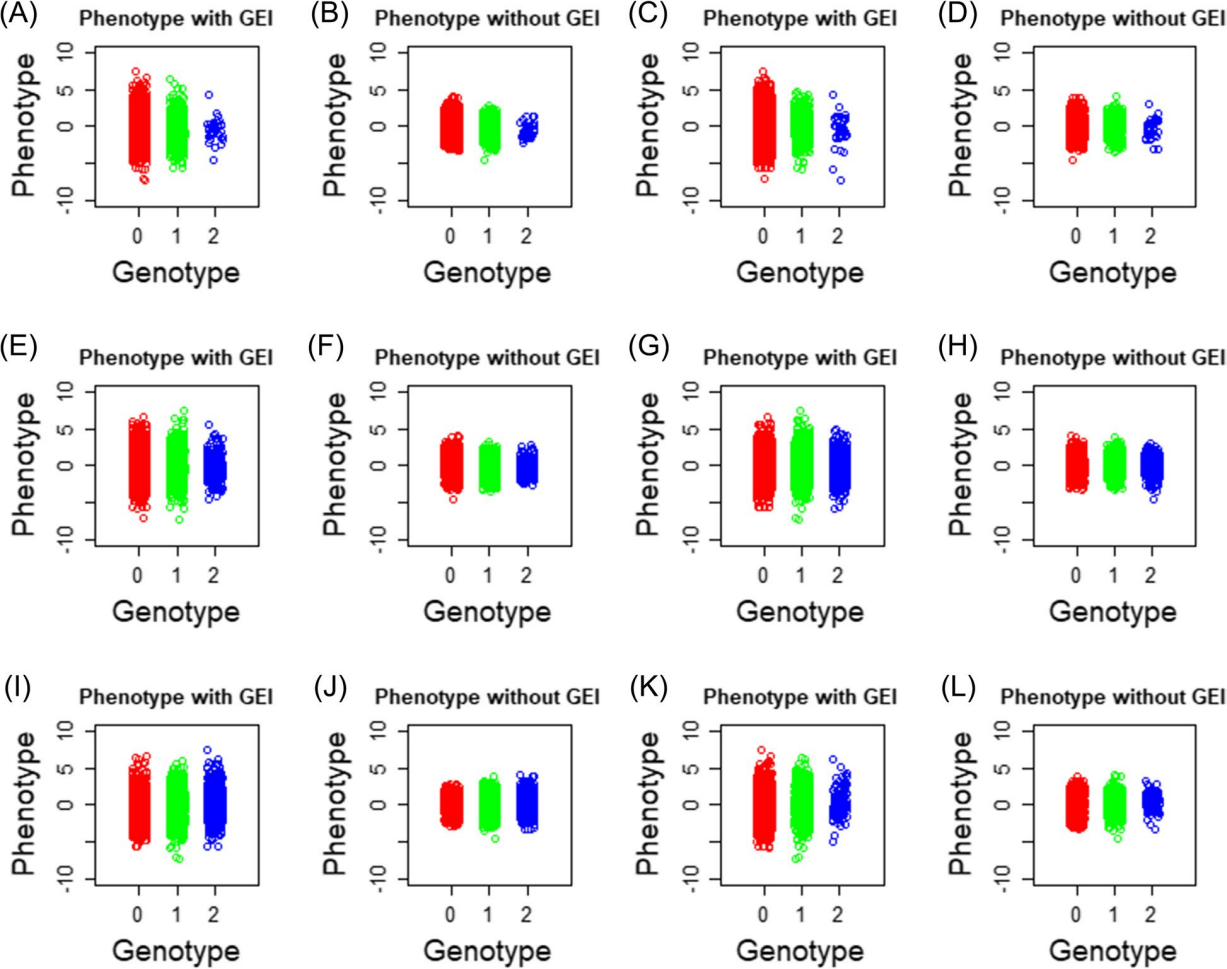
Phenotypic variation for different genotypes of 6 randomly selected SNPs in one replicate of simulated data with or without the G-by-E interaction (GEI). **A** The phenotypic values of individuals with three genotypes of the first SNP with GEI, **B** the phenotypic values of individuals with three genotypes of the first SNP without GEI; **C** the second SNP with GEI, **D** the second SNP without GEI; **E** the third SNP with GEI, **F** the third SNP without GEI; **G** the fourth SNP with GEI, **H** the fourth SNP without GEI; **I** the fifth SNP with GEI, **J** the fifth SNP without GEI; **K** the sixth SNP with GEI, (L) the sixth SNP without GEI

In this section, we generate simulated data considering a G-by-E interaction to assess GPOPSIM2.0. We set one continuous trait and one environmental factor to simulate a G-by-E interaction. The heritability was 0.1, and the additive variance of the interaction (${\sigma}_{\alpha_1}^2$) was 0.25. Twenty random seeds were used to produce 20 replicates of simulation. According to the results of one replicate of simulation (10,000 individuals), the phenotypes and environmental values followed normal distributions (Fig. 2). The phenotypic variation for genotypes of 6 randomly selected SNPs in the data accounting for the G-by-E interaction was much larger than that without the G-by-E interaction (Fig. 3). We used the software DMU [20] to estimate ${\sigma}_{\alpha_0}^2$, ${\sigma}_{\alpha_1}^2$, ${\sigma}_{\alpha_0{\alpha}_1}$ and ${\sigma}_{e_0}^2$ with the reaction norm model with pedigree information (A matrix) and genomic information (G matrix). As shown in Table 1, these estimates were close to the assigned values. As expected, we obtained better estimates using the G matrix than using the A matrix because genomic information can more accurately estimate the relationships between individuals. All of the above results indicate that GPOPSIM2.0 is an ideal tool for simulating G-by-E interactions.

**Table 1:** The assigned and estimated G-by-E parameters in 20 replicates of simulated data from GPOPSIM2.0

Parameter	Assigned	Estimates(A)	Estimates(G)
Var(a_0_)	1	0.702(0.13)	0.943(0.117)
Cov(a_0_,a_1_)	0.026(0.059)^*^	0.033(0.113)	0.011(0.028)
Var(a_1_)	0.25	0.341(0.045)	0.239(0.028)
Var(e_0_)	9	8.828(0.148)	9.076(0.124)

Assigned: parameters set in the program; Estimates (A): estimated by using a reaction norm model with pedigree information; Estimates (G): estimated by using a reaction norm model with genomic information

^*^ Cov(a_0_,a_1_)= ∑2 ∗ *p*_*i*_ ∗ (1 − *p*_*i*_) ∗ *m*_*i*_ ∗ *n*_*i*_, where *p*_*i*_ is the frequency of one allele of the i^th^ QTL, *m*_*i*_ is the effect of the i^th^ QTL for *α*_0_, and *n*_*i*_ is the effect of the i^th^ QTL for *α*_1_

**Fig. 4:**
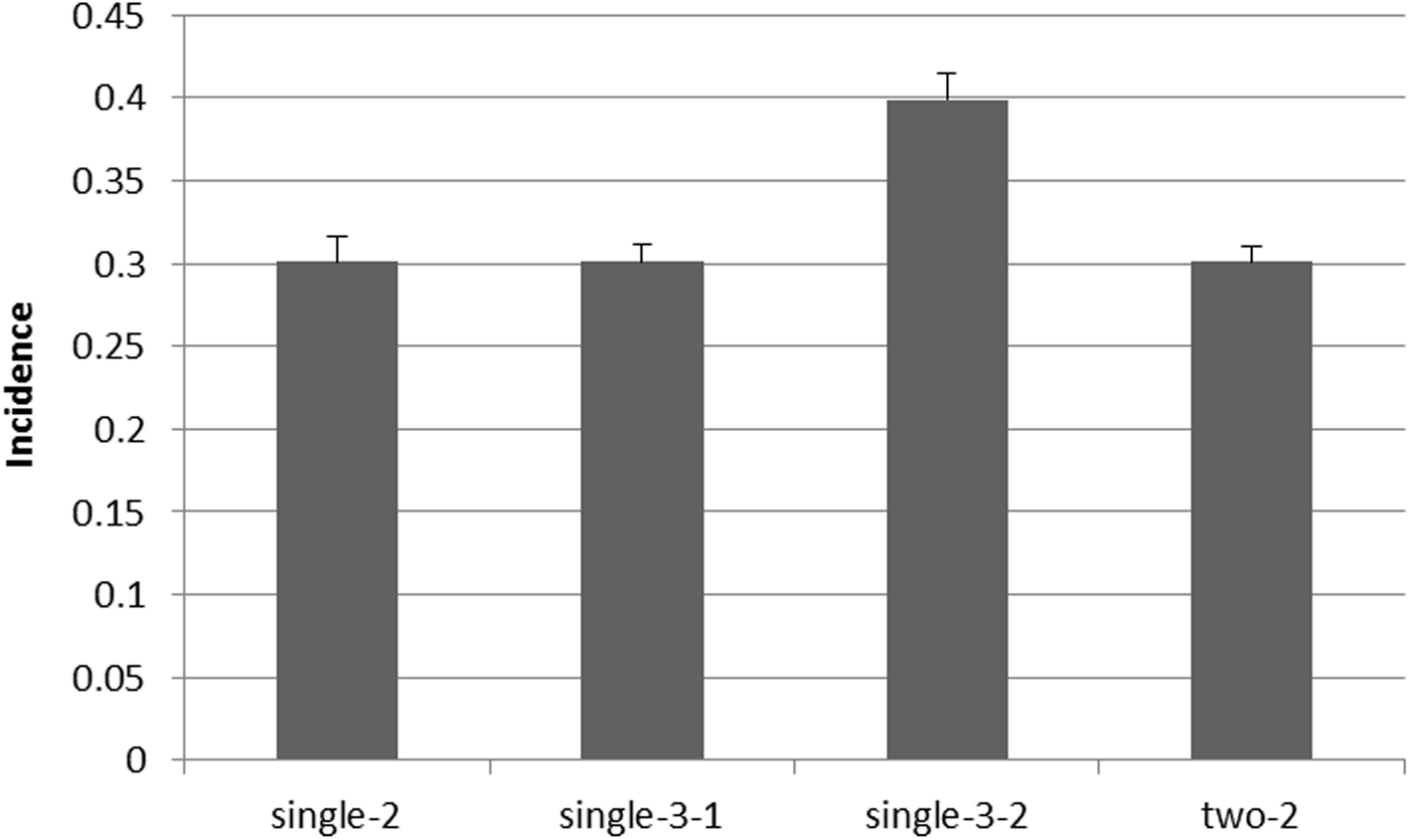
Estimates of the incidence from threshold trait data by GPOPSIM2.0 for 20 replicates. Single-2: one binary trait with an incidence of 0.3; single-3-1: one three-category trait with an incidence of 0.3 for the first category; single-3-2: one three-category trait with an incidence of 0.4 for the second category; two-2: binary- quantitative traits with an incidence of 0.3

Additionally, GPOPSIM2.0 can generate good-quality simulated data for threshold traits. The incidences calculated from simulated data were very close to the set incidences (30% or 40%) from Fig. 4. The estimates (mean ± SD) of incidences were 0.301 ± 0.015 for the binary trait, 0.301 ± 0.009 for the binary-quantitative traits, and 0.301 ± 0.010 and 0.400 ± 0.016 for the three-category traits. These estimates were not significantly different from 30 and 40%, respectively (*P* > 0.05), according to a two-sample t test.

## Conclusions

According to the validation of simulated data, GPOPSIM2.0 has successful new functions for simulating genomic data for G-by-E interactions and threshold traits. GPOPSIM2.0 is a user-friendly tool for simulating large-scale genomic data, and these new functions will aid in the development of new approaches for analysing G-by-E interactions and threshold traits within the framework of GS and GWASs. Nevertheless, there is still room for further improvement of GPOPSIM2.0, such as accommodating QTL epistatic effects and longitudinal genomic data.

## Availability and requirements

Project name: GPOPSIM2.0.

Project home page: https://github.com/SCAU-AnimalGenetics/GPOPSIMv2

Operating system(s): Compiled for Windows and Linux.

Programming language: Fortran 90.

Other requirements: None.

License: None.

Any restrictions to use by non-academics: None.

## Data Availability

The datasets used and/or analysed during the current study are available from the website https://github.com/SCAU-AnimalGenetics/GPOPSIMv2/paper_data.

## Abbreviations

SNP: Single nucleotide polymorphism
QTLs: Quantitative trait loci
GWASs: Genome-wide association studies
GS: Genomic selection
G-by-E interactions: Genotype-by-environment interactions
TBV: True breeding value
